# Science to Practice: Translating Automated Brain MRI Volumetry in Alzheimer’s Disease from Research to Routine Diagnostic Use in the Work-Up of Dementia

**DOI:** 10.3389/fneur.2013.00216

**Published:** 2014-01-09

**Authors:** Bharath Gopal Rathakrishnan, P. Murali Doraiswamy, Jeffrey R. Petrella

**Affiliations:** ^1^Duke University School of Medicine, Durham, NC, USA; ^2^Department of Psychiatry, Duke Institute for Brain Sciences, Duke University Medical Center, Durham, NC, USA; ^3^Department of Radiology, Duke University Medical Center, Durham, NC, USA

**Keywords:** hippocampal volumetry, dementia, Alzheimer’s disease, medial temporal lobe, medial temporal lobe atrophy, volumetrics

The medial temporal lobe (MTL) has long been identified as a key region affected by neurodegenerative diseases, a common source of cognitive decline in the elderly. The importance of the MTL with respect to cognitive decline is furthered by the hippocampus’s role in memory formation (1). Impairment of declarative memory is one of the earliest symptoms of Alzheimer’s disease (AD), the most common form of dementia. Early involvement of the MTL in AD is characterized by the presence of neurofibrillary tangles in the entorhinal cortex. As the disease progresses, neurofibrillary tangles continue to accumulate and involve the hippocampus, amygdala, and ultimately the neocortex (2). This gradual progression mirrors the worsening symptoms patients with AD experience. While initial concerns include memory deterioration, patients in later stages of AD can present with progressive aphasia (3), visuospatial impairments including agnosia (4), and apraxia (5).

Neuroimaging findings from Alzheimer’s patients are consistent with the pathologic and clinical pattern, suggesting potential clinical diagnostic use. Structural magnetic resonance (MR) imaging analysis of patients with mild cognitive impairment (MCI) and AD is most notable for MTL atrophy, including significant volume reduction in the hippocampus, amygdala, parahippocampal gyrus, and entorhinal cortex (6). Structural MRI has identified the entorhinal cortex as the first region to display atrophy in AD. Atrophy in this region is followed by atrophy in the hippocampus, amygdala, and parahippocampal gyrus (7). The next region to display atrophy includes the posterior cingulate cortex; the atrophy then becomes generalized to the temporal neocortex and neocortical association areas (8). Thus, the evolution of neuroimaging findings on MRI closely mirrors the progression of neurodegenerative pathology, suggesting that volume assessment by MRI may be a useful clinical tool for early diagnosis and disease monitoring.

Qualitative visual assessment of temporal lobe atrophy on MRI has demonstrated a sensitivity of approximately 80% in distinguishing AD from normal aging, depression, and vascular dementia (9). A recent study revealed that qualitative visual assessment of MTL atrophy differentiated pathologically confirmed AD from vascular cognitive impairment and dementia with Lewy bodies with a sensitivity of 91% and specificity of 94% (10). Atrophy of the MTL is particularly important because it can be detected earlier than generalized whole brain atrophy in AD. Unfortunately, there is significant variation in qualitative assessment of MTL atrophy among radiologists. One investigation of cognitively normal elderly individuals revealed a 70% interobserver agreement (kappa values of 0.59 and 0.62) in identifying the presence of MTL atrophy on coronal T1-weighted MRI scans (11). Another study included elderly patients with dementia and the interobserver agreement dropped to 49.7%, with kappa values of 0.34 and 0.24 (12). Such poor inter-rater reliability is a major limitation for implementing qualitative MTL atrophy assessments in a clinical setting on individual patients.

This limitation has been addressed by quantitative volumetric analysis of MTL structures, which offers improved reliability and predictive accuracy. A comparison of qualitative ratings of MTL atrophy and hippocampal volumetry revealed that volumetry was more accurate in predicting cognitive decline to AD in MCI patients (100 vs. 78% positive predictive value, 100 vs. 87% negative predictive value) (13). A challenge of using any MTL atrophy assessment is that these structures often display volume loss in cognitively normal patients, much of which is attributed to the normal aging process. However, the effect of AD on MTL atrophy is much greater. Even patients with mild AD display significantly greater volume loss in MTL structures when compared to cognitively normal controls (14). The specific structure within the MTL that has demonstrated the most accuracy in predicting the presence of AD is the hippocampus. A semi-automated tracing-threshold technique for segmenting the hippocampus yielded a sensitivity of 82% and specificity of 80% in identifying patients with AD as compared to controls (14). Serial volume measurements tracking hippocampal change over time show even greater effects compared to baseline values alone. Rates of hippocampal atrophy were found to be considerably greater in Alzheimer’s patients (−3.98%, −150 mm^3^/year) as compared to control patients (−1.55%, −75 mm^3^/year) (15). Additionally, hippocampal atrophy rates were found to correlate with patients’ baseline cognitive statuses and also matched cognitive decline, with atrophy rates increasing from controls (1.73% for stable, 2.81% for decliners) to MCI (2.55% for stable, 3.69% for decliners) to AD (3.5%) (16).

While studies have established this clear finding of hippocampal atrophy in AD, hippocampal volumetry has not yet become a routine part of the diagnostic work-up for neurodegenerative diseases. Volumetric calculations required time-consuming, labor-intensive manual tracing over the borders of MTL structures over multiple slices. Summed regions of interest over the slices are used to count the number of voxels and ultimately produce a volume measurement. Despite the advantages over visual ratings, this method requires individual segmentation of structures within the MTL which may also vary considerably across different operators, depending upon their training and experience. Moreover, it is unclear how to account for the aging process and differences in head size in interpretation of such volume measurements in individual patients.

These concerns have been addressed through the development of software programs such as FreeSurfer, Individual Brain Atlases using statistical parametric mapping (IBASPM), and NeuroQuant that allow for completely automated, user-independent calculations of neural volumes. NeuroQuant, a commercial implementation of the FreeSurfer algorithm, also generates an age-related atrophy percentile, in addition to a general morphometry report (Figure 1). The age-related atrophy report includes the volume, percentage of intracranial volume, and normative percentiles for the hippocampus, lateral ventricles, and inferior lateral ventricles (temporal horns). The normative percentiles are based on control subjects from the multicenter AD Neuroimaging Initiative database (17). Implementations of these programs operate without user input and allow for rapid volumetric calculation and report generation immediately after a patient’s MRI scan has been obtained, making them well-suited for incorporation into a busy clinical workflow.

**Figure 1:**
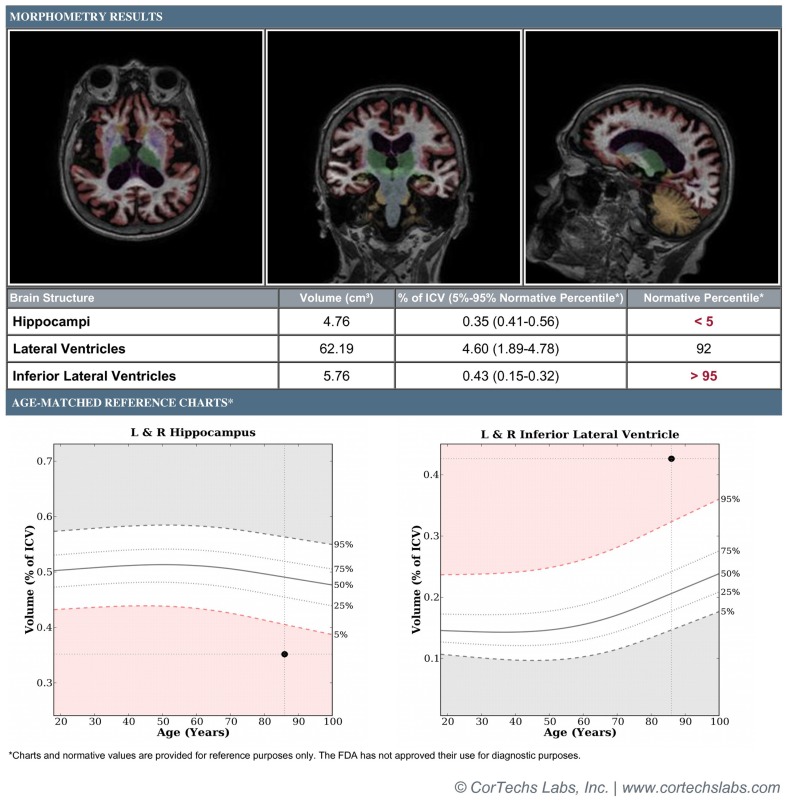
**Patient is an 85-year-old female with a 3-year history of memory loss with differential diagnosis of normal aging vs. mild Alzheimer’s disease vs. vascular dementia**. Figure demonstrates morphometry results displaying axial, coronal, and sagittal color-segmented images of the brain as well as volumetrics expressed in absolute and normalized units. Graphs demonstrate normalized volumes plotted against percentiles for age for the hippocampi and temporal horns. Results demonstrate hippocampal volume below the 5th percentile and temporal horns above the 95th percentile, supporting provisional diagnosis of mild Alzheimer’s disease.

Several of these software packages have been validated in studies comparing Alzheimer’s patients to cognitively normal controls. Volumetric analysis was performed in both groups of patients using NeuroQuant vs. semi-manual segmentation as a gold standard. There was a high degree of agreement in the volumes generated (hippocampus, ICC = 0.93, inferior lateral ventricle, ICC = 0.92). Both methods demonstrated volume loss of the hippocampus and associated increased inferior lateral ventricle size in Alzheimer’s patients as compared to controls (18). FreeSurfer has also been incorporated in assessment of temporal lobe structures in patients with AD and semantic dementia. One investigation revealed that FreeSurfer correlated highly with manual volumetric delineation, particularly in the ventricles (*r* = 0.99), right medial-inferior temporal gyrus (*r* = 0.84), and left hippocampus (*r* = 0.76) (19).

Further investigations are needed to elucidate the clinical role and utility of automated hippocampal volumetry. While studies have demonstrated the sensitivity of hippocampal and MTL atrophy for conditions such as MCI and AD, hippocampal atrophy can occur in a number of other settings. Hippocampal atrophy has been documented in numerous conditions ranging from Cushing’s syndrome, major depression, posttraumatic stress disorder (20), Type 2 Diabetes (21), traumatic brain injury, and concussions (22). Research has also implicated several genotypes associated with hippocampal volumes. Val66Met polymorphisms of the BDNF gene have been linked to worsened episodic memory (23) and reduced hippocampal volumes (24), suggesting BDNF’s role in vulnerability to neural changes associated with aging (25). The APOE ε4 allele has also demonstrated an association with increased rate (26) and amount (27) of hippocampal atrophy. Decreased hippocampal volumes in patients with depression were linked to 44-base pair insertions in the promoter region of the serotonin transporter (5-HTTLPR) (28). Therefore, additional work is needed to establish the specificity of hippocampal atrophy in a general practice setting, including patients with multiple, sometimes coexisting, conditions, and genotypes.

In addition, while we believe hippocampal volumetry can be incorporated into neuroimaging evaluation of patients with potential neurodegenerative diseases, significant work still needs to be done to establish standardized volumetry thresholds for atrophy rates and age-adjusted volumes that will be useful in practice for different clinical contexts, such as differential diagnosis, early identification/prediction, and disease monitoring. A clear delineation of hippocampal boundaries is required along with a standardized, accessible database, and uniform methods of reporting hippocampal volumes (29). Hippocampal volumetry still awaits U.S. FDA approval for specific clinical contexts of use because of the current lack of standardized volumetry norms. Nevertheless, the European Medicines Agency has approved the use of this technique for sample enrichment in clinical trials (30). Though full incorporation in routine practice awaits such norms, complete hierarchical validation (31) may unnecessarily delay implementation; therefore, alternative approaches should be considered for concurrent assessment and implementation of this technology (32). Hippocampal volumetrics may currently be helpful as an aid in differential diagnosis of dementia and MCI in specific cases, particularly when values fall in the normal range. Hippocampal volumetry may be particularly important in identifying patients in the pre-clinical stages of AD and also in predicting worsening of MCI patients. Indeed, new diagnostic criteria have been proposed for both AD and MCI due to AD which incorporate MRI volumetry as a neuronal loss marker (33). Thus, the time is now for hippocampal volumetry to become a component of the neuroimaging evaluation of patients with suspected neurodegenerative disease.

## Conflict of Interest Statement

The authors declare that the research was conducted in the absence of any commercial or financial relationships that could be construed as a potential conflict of interest.
